# Tumor-Derived cGAMP Regulates Activation of the Vasculature

**DOI:** 10.3389/fimmu.2020.02090

**Published:** 2020-09-04

**Authors:** Marco Campisi, Shriram K. Sundararaman, Sarah E. Shelton, Erik H. Knelson, Navin R. Mahadevan, Ryohei Yoshida, Tetsuo Tani, Elena Ivanova, Israel Cañadas, Tatsuya Osaki, Sharon Wei Ling Lee, Tran Thai, Saemi Han, Brandon P. Piel, Sean Gilhooley, Cloud P. Paweletz, Valeria Chiono, Roger D. Kamm, Shunsuke Kitajima, David A. Barbie

**Affiliations:** ^1^Department of Mechanical and Aerospace Engineering, Politecnico di Torino, Turin, Italy; ^2^Department of Medical Oncology, Dana–Farber Cancer Institute, Boston, MA, United States; ^3^University of Virginia School of Medicine, University of Virginia, Charlottesville, VA, United States; ^4^Department of Biological Engineering, Massachusetts Institute of Technology, Cambridge, MA, United States; ^5^Department of Pathology, Brigham and Women’s Hospital, Boston, MA, United States; ^6^Belfer Center for Applied Cancer Science, Dana-Farber Cancer Institute, Boston, MA, United States; ^7^Blood Cell Development and Function Program, Fox Chase Cancer Center, Philadelphia, PA, United States; ^8^Department of Mechanical Engineering, Massachusetts Institute of Technology, Cambridge, MA, United States; ^9^Institute of Industrial Science, The University of Tokyo, Tokyo, Japan; ^10^Singapore-MIT Alliance for Research & Technology, BioSystems and Micromechanics, Singapore, Singapore; ^11^Department of Microbiology and Immunology, Yong Loo Lin School of Medicine, National University of Singapore, Singapore, Singapore; ^12^Department of Cell Biology, Cancer Institute, Japanese Foundation for Cancer Research, Tokyo, Japan

**Keywords:** LKB1, 2′3′-cGAMP, STING, KRAS, T cell, endothelial cells, microfluidic culture

## Abstract

Intratumoral recruitment of immune cells following innate immune activation is critical for anti-tumor immunity and involves cytosolic dsDNA sensing by the cGAS/STING pathway. We have previously shown that KRAS-LKB1 (KL) mutant lung cancer, which is resistant to PD-1 blockade, exhibits silencing of STING, impaired tumor cell production of immune chemoattractants, and T cell exclusion. Since the vasculature is also a critical gatekeeper of immune cell infiltration into tumors, we developed a novel microfluidic model to study KL tumor-vascular interactions. Notably, dsDNA priming of LKB1-reconstituted tumor cells activates the microvasculature, even when tumor cell STING is deleted. cGAS-driven extracellular export of 2′3′ cGAMP by cancer cells activates STING signaling in endothelial cells and cooperates with type 1 interferon to increase vascular permeability and expression of E selectin, VCAM-1, and ICAM-1 and T cell adhesion to the endothelium. Thus, tumor cell cGAS-STING signaling not only produces T cell chemoattractants, but also primes tumor vasculature for immune cell escape.

## Introduction

Immune recognition of tumor cells in the tumor microenvironment (TME) requires effector T cells and other immune cells to extravasate from the vasculature and migrate through the extracellular matrix (ECM) to recognize tumor antigens. Indeed, resistance to PD-1 immune checkpoint blockade (ICB) has been linked to an “immune cell excluded” phenotype in many tumor types (1–3). For example, KRAS mutant non-small cell lung cancers that inactivate the STK11/LKB1 tumor suppressor are strongly resistant to anti-PD-(L)1 therapy and exhibit T cell exclusion (4, 5).

Stimulation of Interferon Genes (STING), an ER-resident protein encoded by *TMEM173*, is an important mediator of the innate immune response to pathogens and in cancer (5–10). Cyclic GMP-AMP synthase (cGAS) recognizes double-stranded DNA (dsDNA) in the cytosol and binds it to generate 2′3′-cGAMP, a cyclic dinucleotide and soluble second messenger that binds STING, which causes activation of the kinase TBK1 via phosphorylation and its downstream substrate, the transcription factor IRF3 (8). Thus, phosphorylated TBK1 (pTBK1) and production of specific cytokines downstream of IRF3, such as CXCL10, can be measured as a function of STING activation, which plays important roles in tumor cells, antigen presenting cells, and potentially other cell types (10). Recently our group identified epigenetic silencing of STING in KRAS-LKB1 (KL) mutant cancer cells, due to an autophagic defect and consequent cytosolic accumulation of mitochondrial DNA, resulting in the inhibition of STING-TBK1-IRF3 mediated type I interferon signaling and impaired production of T cell chemoattractants such as CXCL10 (5). These findings were supported by *in vivo* quantitative IHC data from patient biopsies that demonstrated impaired intratumoral T-cell infiltration from KL tumors lacking STING expression, and instead, retention of T cells in the stroma (5). STING silencing has also been reported in other tumor types with high tumor mutational burden (TMB) such as melanoma, where loss of STING also mediates escape from recognition of tumor antigens (11).

Communication between cancer cells and the vasculature can modulate infiltration of immune cells and regulate the composition of the TME, though the role of cGAS-STING signaling in this process has not been characterized (12). Cancer cells are known to communicate with neighboring cells, such as astrocytes in the brain TME, which can activate STING via 2′3′-cGAMP in a paracrine manner and promote metastasis (7). Emerging work also reveals that tumor derived 2′3′-cGAMP can act as an immunotransmitter and directly influence anti-tumor immunity (8, 13, 14). Given the problem of immune cell exclusion in many tumor types there is an increasing need to understand how the subcomponents of the TME and especially the tumor vasculature regulates immune extravasation. Importantly, tumor vascular endothelial cells have been identified as a major source of type 1 interferon production in the TME following intratumoral injection of 2′3′-cGAMP-based STING agonists, which promote T-cell-mediated therapeutic antitumor immunity (15). These studies suggest that endogenous 2′3′-cGAMP could also influence the tumor vasculature and regulate its activation in a paracrine manner.

We have also previously reported the use of microfluidic devices to support 3-dimensional (3-D) culture of perfusable microvascular networks (MVNs), comprised of human umbilical vein endothelial cells (HUVECs) and human lung fibroblasts (hLFBs) in a supportive ECM-like gel (collagen or fibrin), which self-organize into vasculature after 5 days of co-culture (16, 17). The same microfluidic devices also enable 3-D culture of cancer cells in similar hydrogels using tumor cell aggregates (spheroids) previously formed in ultra-low attachment plates for 24 h (18–20). This system enables more detailed study of the biological interactions between KRAS mutant cancer cells and the tumor microvasculature. Thus, we developed a microfluidic model that could support culture and formation of vascularized KL lung cancer spheroids, with the specific goal of studying how tumor cell dsDNA sensing via cGAS-STING might modulate innate immune signaling in this more physiologically relevant milieu.

## Materials and Methods

### Immunohistochemical (IHC) Staining and Data Analysis

Brain tumor and brain metastasis tissue microarrays (GL2082, GL861) were purchased from US Biomax, Inc and IHC was performed on the Leica Bond III automated staining platform. The antibody for phospho-TBK1 (Cell Signaling Technology #5483, clone D52C2) was run at 1:50 dilution using the Leica Biosystems Refine Detection Kit with EDTA antigen retrieval. The antibody for STING (Cell Signaling Technology #13647, clone D2P2F) was run at 1:50 dilution using the Leica Biosystems Refine Detection Kit with citrate antigen retrieval. Staining was visually scored in a binary manner (presence/absence) in endothelial cells identified using the hematoxylin counterstain marking a circumferential layer of nuclei surrounding red blood cell fragments. These results were confirmed by a board-certified anatomic pathologist (NRM), who also quantified infiltrating lymphocytes by morphology on hematoxylin-counterstained per high power field (HPF = 40× objective), averaged across confidently identified endothelial lumens in 1–4 HPF per specimen. Average tumor infiltrating lymphocytes per HPF was compared for pTBK1+ and pTBK1− blood vessels in each tumor specimen.

### Cell Lines

H1355 were cultured in RPMI-1640 (Thermo Fisher Scientific, Cat.# 11875-119) supplemented with 10% FBS, 1× penicillin–streptomycin, and 2.5 (g/mL plasmocin prophylactic (InvivoGen, Cat.# ant-mpp). Cells were originally obtained from the Broad Institute and authenticated by short tandem repeat genotyping. Human umbilical vein endothelial cells, HUVECs (Lonza, C2519AS) were cultured in vascular medium (VascuLife^®^ VEGF Endothelial Medium Complete Kit, #LL-0003). NHLF- human Lung Fibroblasts (Lonza, CC-2512) were cultured in fibroblast growth supportive medium (FibroLife^®^ S2 Fibroblast Medium Complete Kit, # LL-0011). Culture medium was replaced every 2 days, and all experiments were performed before reaching 10 passages. Mycoplasma infection was regularly checked by PCR using the conditioned media derived from each cell line. The sequences of the primers used for checking mycoplasma infection are listed in Supplementary Table S1.

### CRISPR/Cas9 System

Target sequences for CRISPR interference were designed using the single-guide RNA (sgRNA) designer^1^. A non-targeting sgRNA from the Gecko library v2 was used as a scramble sgRNA. sgRNA target sequences are listed in Supplementary Table S1.

### Generation of Lentivirus

HEK293T cells (3 × 10^6^) were plated onto a 60-mm dish and transected using X-tremeGENE HP DNA Transfection Reagent (Roche, Cat.# 06366236001) with 1 μg of lentivirus-based expression vectors together with 1 μg of pCMV-dR8.91 and 1 μg of pCMV-VSV-G. After 48-h incubation, the media containing lentivirus particles were collected, passed through a 0.45 μm filter, and concentrated using Lenti-X Concentrator (Clontech, Cat.# 631231). For selection of virally infected cells, 1 μg/mL of puromycin (pCRISPR-v2 sgRNAs) or 6 μg/mL of blasticidin (plx304-NanoLuc or plx304-hLKB1) was used 24 h after infection.

### dsDNA Stimulation

Cells (2 × 10^5^ to 5 × 10^5^) were plated onto a 6-well plate and transfected using X-tremeGENE HP DNA Transfection Reagent (Roche, Cat.# 06366236001) with the indicated amount of poly (dA:dT) (*Invivo*- Gen, Cat.# tlrl-patn). Cells utilized for 3D culture in microfluidic devices were transfected for 24 h, then transferred into ultra-low attachment dish to form spheroids as described below.

### Immunoblotting

Cells were lysed in RIPA buffer containing 1× protease inhibitors (Roche, Cat.# 11-836-145-001) and phosphatase inhibitors (50 mmol/L NaF and 100 mmol/L Na3VO4). Immunoblotting was performed as previously reported (5). Briefly, protein was isolated from cell lines and measured by BCA (Pierce Biotechnology). Protein extracts were subjected to polyacrylamide gel electrophoresis using the 4–12% NuPAGE gel system (Invitrogen), transferred to PVDF (Millipore) membranes, and immunoblotted using antibodies that specifically recognize STING (#13647, Cell Signaling Technology), cGAS (#15102, Cell Signaling Technology), LKB1 (#3047, Cell Signaling Technology), phospho-TBK1 (#5483, Cell Signaling Technology), TBK1 (#3013, Cell Signaling Technology), and β-actin (#3700, Cell Signaling Technology). Secondary antibodies were from LI-COR Biosciences: IRDye 680LT Goat anti-Mouse IgG (#926-68020) and IRDye 800CW Goat anti-Rabbit IgG (#926- 32211). LICOR blocking buffer (no. 927-40000) was used to dilute primary and secondary antibodies, with the exception of phosho-specific antibodies, which were diluted in HIKARI Signal Enhancer Solutions 1 and 2 (Nacalai United States, Inc., no. NU00101). Imaging of blots and quantitation of bands were performed using the LI-COR Odyssey system.

### Quantitative RT-PCR and PCR Profile Array

Total cellular RNA was extracted using the RNeasy Mini Kit (Qiagen, Cat.# 74106) according to manufacturer’s instructions. RNA samples (1 μg) were reverse-transcribed using Super- Script III First-Strand Synthesis SuperMix (Thermo Fisher Scientific, Cat.# 1683483). Quantitative real-time PCR was performed using Power SYBR Green PCR Master Mix (Thermo Fisher Scientific, Cat.# 4367659), and the Applied Biosystems 7300 Fast real-time PCR system and software. The relative expression was normalized with the expression of the housekeeping gene 36B4. The sequences of the primers used for qRT-PCR are listed in Supplementary Table S1. Values represent the average of four technical replicates from at least two independent experiments (biological replicates). The profile expression of 84 genes related to endothelial cell biology were performed using the RT (2) Profiler PCR Array for human endothelial cell biology (Cat. # PAHS-015ZC, Qiagen), reverse transcribed and quantitative real-time PCR was performed using RT (2) First Strand Kit (Cat.# 330404, Qiagen), QuantiTect Reverse Transcription Kit (Cat.# 205313, Quiagen), RT (2) SYBR Green ROX qPCR Mastermix (Cat.# 330523, Quiagen) and Applied Biosystems 7300 Fast real-time PCR system and software.

### 3-D Microfluidic Device Design and Fabrication

Tumor-vascular interactions were evaluated using a commercial 3-D cell culture chip (DAX-1, AIM Biotech, Singapore) as previously described (16–18, 20) (Supplementary Figure S5A), and custom microfluidic devices composed of poly-dimethylsiloxane (PDMS; Sylgard 184; Dow Corning, MI, United States). Custom microfluidic device design and fabrication was conducted by standard soft-lithography techniques. Briefly, elastomer and curing agents were mixed (10:1 vol ratio), degassed, and poured onto a silicon master and cured overnight at 60°C. Access ports for hydrogel injection and media channels were created with biopsy punches and then the devices were taped to remove dust and sterilized in an autoclave. PDMS devices were treated with oxygen plasma (Harrick Plasma), bonded to a glass coverslip (Fisher Scientific) and finally placed in oven until use. Micro-devices have a central gel channel 2200 μm wide and 150 μm high, flanked by two medium channel 1340 μm wide (Supplementary Figure S5B). Macro-devices have a central gel channel 3 mm wide and 0.5 mm high, flanked by two fluidic channels 3 mm wide (Supplementary Figure S5C). 3-D cell culture chips and micro-devices were used for biological cellular studies. Macro-devices were used for permeability measurements.

### 3-D Microfluidic Culture

Cancer cell spheroids were generated by seeding 5 × 10^5^ cells in suspension in an ultra-low attachment dish (Corning, Cat.# 3471) for 24 h. Samples were pelleted and resuspended in type I rat tail collagen (Corning) at a concentration of 2.5 mg/mL following the addition of 10 × PBS with phenol red with pH adjusted using NaOH. pH of 7.0 to 7.5 was confirmed using PANPEHA Whatman paper (Sigma-Aldrich). All pelleted spheroids were resuspended in 250 μL of collagen hydrogel. The spheroid-collagen suspension was then injected into the central gel region of the 3D microfluidic device. After injection, devices containing spheroid-collagen mixture were incubated for 40 min at 37°C in humidity chambers, then hydrated with culture media, and refreshed daily for 7 days.

Microvascular networks (MVN) were created by detaching HUVEC and hLFB cells from cell culture flasks and resuspending them in cold vascular medium (Vasculife, Lifeline #LL-0003) with 2 U/ml thrombin from bovine plasma (Millipore Sigma, #T7326). The two cell types were combined with cell densities of 12 × 10^6^/ml HUVECs and 2 × 10^6^/ml hLFB. Cell suspensions were mixed 1:1 volume ratio with 6 mg/ml fibrinogen (Millipore Sigma, #341573) and gently injected into microfluidic devices. After allowing several minutes of fibrin polymerization (15–30 min) in a 37°C incubator, warm vascular medium was added to the flanking media channels and refreshed each day of culture. MVN self-assembled over several days refreshing media daily. To generate the MVN + Spheroids samples, MVN were co-cultured with tumor spheroids and protocols were combined. To maintain the same conditions used in each individual protocol, cell densities of 24 × 10^6^/ml HUVECs and 4 × 10^6^/ml hLFB were mixed 1:1 volume ratio with 6 mg/ml fibrinogen. This cell-gel suspension was mixed 1:1 with collagen-spheroids mixture previously generated by resuspending spheroids in 125 μL of collagen hydrogel, resulting in the same final cell density as MVNs or Spheroids alone. To maintain consistency for cytokine analysis, all microfluidic devices conditions were cultured in vascular medium (Vasculife, Lifeline #LL-0003).

MVN alone in microfluidic device were treated with 1 μg/ml 2′3′-cGAMP, or 100 ng/ml IFN-β, or both in combination after 6 days of culture, after which conditioned medium was collected or device were imaged with confocal microscopy 24 h later.

### 3D Permeability Measurements and Quantification

To measure the vascular permeability of fluorescent dextran in 3D, MVN alone in microfluidic device were cultured for 7 days and treated with 1 μg/ml 2′3′-cGAMP or 100 ng/ml IFN-β. Firstly, medium was removed from both media channels of a macro-device. A solution of fluorescein isothiocyanate (FITC)-conjugated dextran (Sigma-Aldrich) (70 kDa, 0.1 mg/ml) was added to each media channel in succession, and the device was transferred to a confocal microscope (Olympus FV1000). Three regions of interest (ROI) were chosen randomly along the gel channel to ensure non-biased sampling of the microvasculature, and z-stacks approximately 50 μm high were acquired immediately after addition of the fluorescent tracer and 15 min later (512 × 512 pixels, 20× magnification). Microvascular permeability was quantified by considering the increase in fluorescence intensity of FITC-dextran within the extra-vascular gel region as previously described (21). Briefly, Vascular network permeability, *P*, was quantified by measuring the average fluorescence intensity of the vascular (*I*_*v*_) and matrix (*I*_*m*_) compartments at two different time points *t*_1_ and *t*_2_ (*t_2_ − t_1_* = Δ*t*):

$$P=\frac{V_{m}}{S\,A\,\Delta \,I}\,\frac{\Delta \,I_{m}}{\Delta \,t}$$

Δ*I_*m*_* = *I_*m*,__2_ – I_*m*,__1_* is the increase in mean fluorescence intensity, in the matrix of volume *V*_*m*_ between time points and Δ*I* = *I_*v*,__1_ − I_*m*,__1_*, is the difference in fluorescence intensity, therefore solute concentration, between the vasculature (with surface area *SA*) and matrix at the start of the measurement. Reconstruction and segmentation was performed with Fiji (22) using the 3D Trainable Weka Segmentation plugin for quantification of parameters such as surface area (SA) and volume of the vascular network and matrix. The fluorescent intensity values were computed using Fiji.

### 3D Perfusion and Adhesion Assay

To test adhesion of T cells to the vascular endothelium, Jurkat T cells were perfused through microvascular networks on day 7 after loading the microfluidic devices. Jurkat cells (Clone E6, ATCC TIB-152) were cultured in RPMI-1640 medium with 10% FBS and 1% penicillin–streptomycin and dyed with CellTracker Green CMFDA Dye (Thermo Fisher Scientific) before experiments. Half of the networks were treated with 1 μg/ml of 2′3′-cGAMP in vascular medium on day 6, and incubated for 24 h. On day 7 devices were perfused with fresh medium and then incubated with sterile rhodamine Ulex Europaeus Agglutinin I (Vector Laboratories) for 20 min to label the endothelium and washed again with fresh vascular medium before introducing Jurkat T cells. The Jurkat cells were pelleted and suspended in vascular medium at 106 cells/ml. Each device received 40 μl of the cell suspension in one channel of the microfluidic device, and cells were allowed to flow through the vascular networks for 30 min before perfusing fresh medium to wash away unbound cells. Jurkat cells that remained bound to the vascular networks were imaged with confocal microscopy (Olympus FV1000) and counted in FIJI. The number of cells retained in untreated networks and those treated with 2′3′-cGAMP were compared using a 2-sided student’s *T*-test.

### Immunofluorescence and Confocal Imaging

Mature microvascular networks were rinsed with warm PBS followed by the addition of approximately 100 μl of 4% paraformaldehyde (Electron Microscopy Sciences, # 15700) to the media channels and left at room temperature. After 15 min of fixation, devices were rinsed twice with PBS, and blocking solution (4% bovine serum albumin, 0.5% goat serum) (Sigma-Aldrich) was added. Devices were incubated for 1 day at 4°C, washed with PBS, and stained with primary antibodies: ICAM-1 (Biolegend, 4453320),VCAM-1 (Abcam, ab134047), CD31 (Abcam, ab28364), conjugated Alexa Fluor 647 anti-human CD326 (EPCAM) (BioLegend, 324212), Acti-stain 555 phalloidin, F-actin (Cytoskeleton, PHDH1-A) and incubated at 4°C for another day. Devices were again washed with PBS and secondary antibodies (Thermo Fisher Scientific, A-11070, A-11011, A-21052) DAPI (4′,6-Diamidino-2-Phenylindole, Dihydrochloride, Invitrogen) or DyLight 649 labeled Ulex Europaeus Agglutinin I (Vector Laboratories) were added, followed by incubation at 4°C protected from light. Finally, samples were washed again with PBS and 3D images were acquired with a confocal microscope (Olympus FV1000) at 20×. Z-stacks were collapsed with maximum intensity projections for viewing (800 × 800 pixels) using FIJI (22).

### Multiplexed Cytokine/Chemokine Profiling

Multiplexed assays were performed utilizing the bead-based immunoassay approach Bio-Plex Pro Human Cytokine 40-plex Assay (Cat.# 171AK99MR2) on a Bio-Plex 200 system (Bio-Rad Laboratories, Cat.# 171000201) and the Human Cytokine/Chemokine Magnetic Bead Panel (Cat.# HCYTMAG-60K-PX30) on a Luminex MAGPIX system (Merck Millipore). Conditioned media concentration levels (pg/mL) of each protein were derived from 5-parameter curve fitting models. Fold changes relative to the corresponding control were calculated and plotted as log2FC. Lower and upper limits of quantitation (LLOQ/ULOQ) were imputed from standard curves for cytokines above or below detection. The degree of cytokine/chemokine modulation (D) in the MVN + Spheroids co-culture samples was calculated from absolute concentration levels (pg/mL) of the values from isolated MVN culture were subtracted to the MVN + Spheroids co-culture and results normalized to spheroid-only results as represented by the following equation:

$$D=\frac{\left(\left(\text{MVN}+\text{Spheroids}\right)-\text{MVN}\right)}{S\,p\,h\,e\,r\,o\,i\,d\,s}$$

Where the resulted degree cytokine/chemokine modulation is simply additive (D = 1) versus supra-additive (D > 1) or antagonistic (D < 1).

### ELISA

Human IFN-β (Thermo Fisher Scientific, Cat.# 414101), CXCL10 (R&D systems, Cat.# DIP100) and 2′3′-cGAMP (Cayman Chemical, Cat.#501700) were detected with ELISAs according to the manufacturer’s instructions. Conditioned media from each cell line were collected after 24-, 48-, or 168-h culture. Values from 2-D cell culture represent the average of two replicates from at least two independent experiments. Values from 3-D cell culture devices represent the average of four replicates from at least three independent experiments (biological replicates).

### Cell Sorting by CD31

Cells (1 × 10^6^) resuspended in 100 μL PBS containing 3% FBS were stained by APC-conjugated anti–CD31 antibody (R&D Systems, Cat.# FAB3567A-025) for 30 min at room temperature, washed by PBS containing 3% FBS, and then analyzed by FACSCanto ll (BD Biosciences). PE/Cy7-conjugated mouse IgG2b (BioLegend, Cat.# 400325) was used as isotype control antibody. Flow sorting for CD31-positive cells was then confirmed with CD31 gene expression by RT-PCR.

### Statistical Analysis

All data are plotted as mean ± SD. Sample size (*n*) is equal to 2 biological replicates or otherwise stated. Unpaired student’s *t*-test was used for significance testing between two conditions. One-way ANOVA with pairwise comparisons by the Tukey *post hoc* test was used to determine whether three or more data-sets were statistically significant. Statistical tests were performed using PRISM7 (GraphPad software) and R (23). *P* values less than 0.05 were considered significant, ^∗∗^*P* < 0.01, ^∗^0.01 < *P* < 0.05.

## Results

### LKB1 Reconstitution Promotes STING-Driven Cytokine/Chemokine Production in 3-D KL Spheroids

**FIGURE 1:**
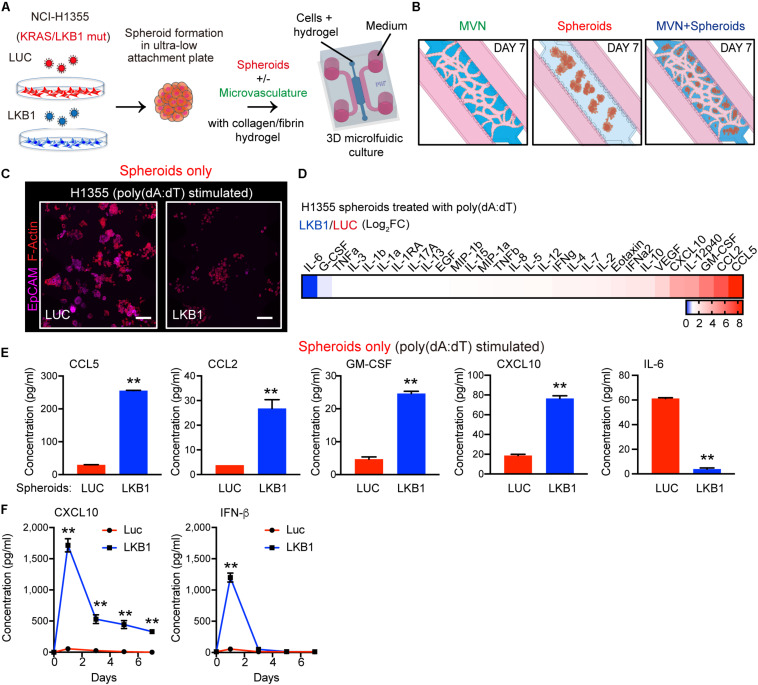
LKB1 reconstitution of 3-D KL spheroids and response to dsDNA in microfluidic culture. **(A)** Schematic of H1355 tumor spheroids formation and their *in vitro* dynamic coculture with or without the microvasculature (MVN) in a 3D microfluidic device within a collagen/fibrin hydrogel. **(B)** Schematic of the dynamic culture of microvasculature only, spheroids only and the combination of spheroids and microvasculature in the microfluidic culture. **(C)** Confocal image of luciferase (LUC) control expressing (left) and LKB1 reconstituted (right) H1355 spheroids in 3D microfluidic culture after 7 days, pre-stimulated with poly 1 μg/mL poly (dA:dT), immunostained for F-actin (red) and EpCAM (CD326) (violet). Scale bar, 150 μm. **(D)** Heat map of cytokine profiles in conditioned medium (CM) 7 days from 3D microfluidic culture of H1355 spheroids. CM was collected 7 days after pre-stimulation with 1 μg/mL poly (dA:dT). Values represent log2 fold change of LKB1 reconstituted H1355 spheroids relative to control. **(E)** Absolute values of cytokine release of human CCL5, CCL2, GM-CSF, CXCL10, and IL-6 produced from 3D microfluidic culture of H1355 LKB1-reconstituted spheroids versus control. **(F)** ELISA of human CXCL10 and IFN-β over 7 days of 2D culture, treated ± 1 μg/mL poly(dA:dT), (*n* = 3 biological replicates). CM was collected and refreshed daily. *P* values were calculated by unpaired two tailed student *t*-test; **P < 0.01. Data shown as mean values, error bars ± SD.

Since LKB1 modulates STING expression in KL cells, and the KL non-small-cell lung cancer (NSCLC) cell line NCI-H1355 (H1355) potently attracts T cells following LKB1 reconstitution in 3-D microfluidic culture (5), we utilized this system to study additional interactions between tumor cell dsDNA sensing and the vasculature. H1355 cells stably expressing a luciferase control (H1355-LUC) or reconstituted with LKB1 (H1355-LKB1) were cultured in ultra-low-attachment dishes for 24 h to allow self-aggregation into 3-D spheroids. Spheroids were cultured in a microfluidic device within a collagen/fibrin hydrogel with or without the self-assembling microvascular network (MVN) using our established HUVEC and hLFB co-culture method (16) (Figures 1A,B). HUVECs self-organize into stable and perfusable MVNs, sustained by hLFBs, which provide paracrine support for MVN formation, reduce the diameter of capillary structures, and produce extracellular matrix (ECM) for stability in long-term culture (16).

Consistent with the tumor suppressive nature of LKB1, H1355-LKB1 spheroids grown after 7 days in microfluidic culture without MVNs exhibited decreased proliferation as compared with H1355-LUC spheroids, but remained viable (Figure 1C). We first validated the direct role of LKB1 in modulating cancer cell-intrinsic dsDNA sensing using both 2-D and 3-D cell culture systems. As expected, introduction of cytoplasmic DNA via poly(dA:dT) transfection (1 μg/ml) followed by continued 2D culture resulted in significantly more robust TBK1 activation following LKB1 reconstitution, consistent with its ability to restore STING expression downstream of AMPK activation (5) (Supplementary Figure S1A). Similarly, poly(dA:dT) transfection of cancer cells followed by spheroid formation and microfluidic 3-D culture revealed that LKB1-reconstituted 3-D spheroids uniquely responded to transfection of cytoplasmic DNA, significantly upregulating multiple immune cell chemoattractants including CCL5, CCL2, and CXCL10, after 7 days (Figures 1D,E and Supplementary Figure S1B). Notably, IL-6 was suppressed by LKB1 reconstitution as previously described (24, 25) (Figure 1E and Supplementary Figure S1B). In contrast, control MVNs expressed high levels of CCL2 and IL-8, as well as IL-6 (Supplementary Figures S1C,D).

Since these studies were conducted at the 7-day endpoint, we next sought to understand the kinetics of cGAS-STING regulated cytokine/chemokine production over time, focusing on CXCL10, the major T cell chemokine downstream of STING and TBK1-IRF3 signaling. We also compared results with IFN-β, another critical TBK1-IRF3 effector cytokine that was not included on the multiplexed array. Notably, poly(dA:dT)-induced IFN-β secretion was also potently restored by LKB1 reconstitution, but fell off by day 3, whereas CXCL10 production was maintained over the 7 day period (Figure 1F) consistent with its known feed-forward activation by IFN (26–28). Thus, LKB1 reconstitution of KL tumor spheroids restores sensitivity to dsDNA sensing in this 3-D culture model.

### A 3-D Microfluidic Co-culture System Captures Changes in Innate Immune Signaling During Tumor-Vasculature Interaction

**FIGURE 2:**
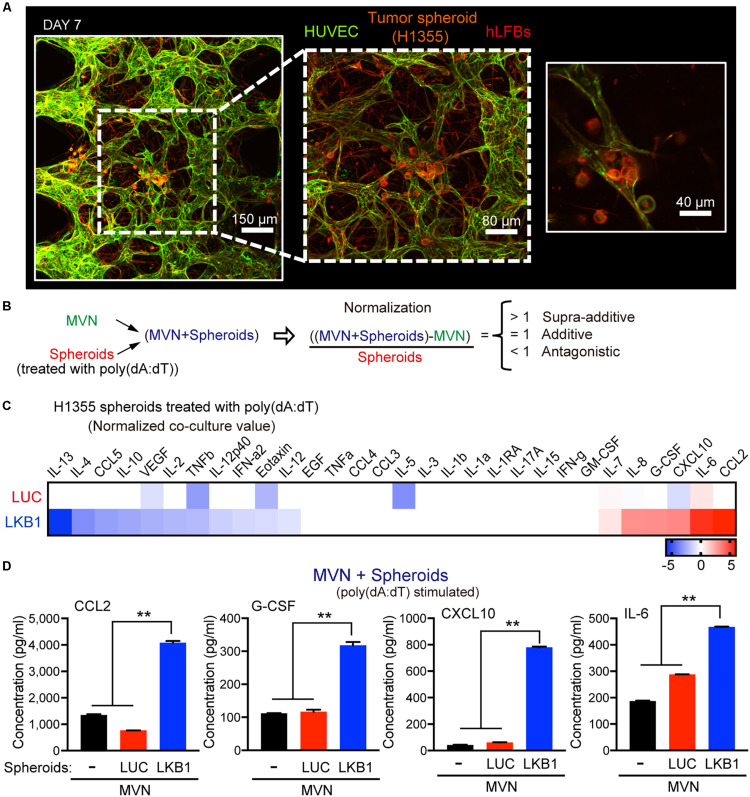
Impact of LKB1 reconstitution on dsDNA sensing in co-culture with MVNs. **(A)** Confocal images at 10×, 20×, and 40× magnification of MVNs formed from HUVECs and hLFBs co-cultured with H1355 tumor spheroids within collagen/fibrin hydrogel after 7 days, immunostained for F-actin (red) and EpCAM (CD326) (orange) and CD31 (green). Scale bars, 150, 80, and 40 μm. **(B)** Schematic equation illustrating normalization of cytokine production from combination of MVN and H1355 tumor spheroids. Cytokine production is considered supra-additive (>1), additive (=1) or antagonistic (<1). **(C)** Heat map of log2 fold change cytokine/chemokine profiles in conditioned medium after 7 days of 3D microfluidic culture of MVN with H1355 LUC and LKB1 spheroids pre-stimulated with 1 μg/mL poly (dA:dT). **(D)** Absolute values of supra-additive cytokine release of human CCL2, G-CSF, CXCL10, and IL-6, from 3D microfluidic culture of combination of MVN + spheroids of H1355 LKB1 and LUC control spheroids and MVN only after 7 days of culture in 3D microfluidic devices. *P* values were calculated by one-way ANOVA followed by Tukey *post hoc* test; **P < 0.01. Data shown as mean values, error bars ± SD.

We next considered the possibility that LKB1 expression in tumor cells might influence innate immune signaling in the neighboring vasculature, especially following dsDNA stimulation of tumor cells. We therefore co-cultured HUVECs/hLFB with H1355 spheroids in microfluidic devices for 7 days, which resulted in effective formation of MVNs and encapsulation of 3D tumor spheroids that are randomly distributed in the perivascular space and in contact interaction with the MVNs (Figure 2A).

To capture differences in dsDNA-stimulated cytokine production that occurred in co-culture with the microvasculature, tumor spheroids were exposed to poly(dA:dT) prior to loading in the device, followed by multiplexed profiling of conditioned media with or without co-culture of MVN with spheroids (H1355-LKB1 vs. LUC). To assess the degree to which the combination of MVN and spheroids modulated cytokine/chemokine production in a simply additive versus supra-additive or antagonistic manner, values from isolated MVN culture were subtracted, and results normalized to spheroid-only results (Figure 2B and Supplementary Figures S1B,D). This calculation revealed supra-additive upregulation of many of the same cytokines/chemokines in LKB1-reconstituted tumor spheroids co-cultured with MVNs, including CCL2 and CXCL10, while there were some differences such as IL-6, which was amplified, and CCL5, which was suppressed (Figures 2C,D). Thus, activation of cGAS-STING signaling by dsDNA in tumor cells alone strongly cooperated with MVNs to amplify innate immune production of specific cytokines and chemokines.

### Cooperative Production of Innate Immune Cytokines Induced by Tumor-Vasculature Interaction Is Not Dependent on Cancer Cell Intrinsic STING

**FIGURE 3:**
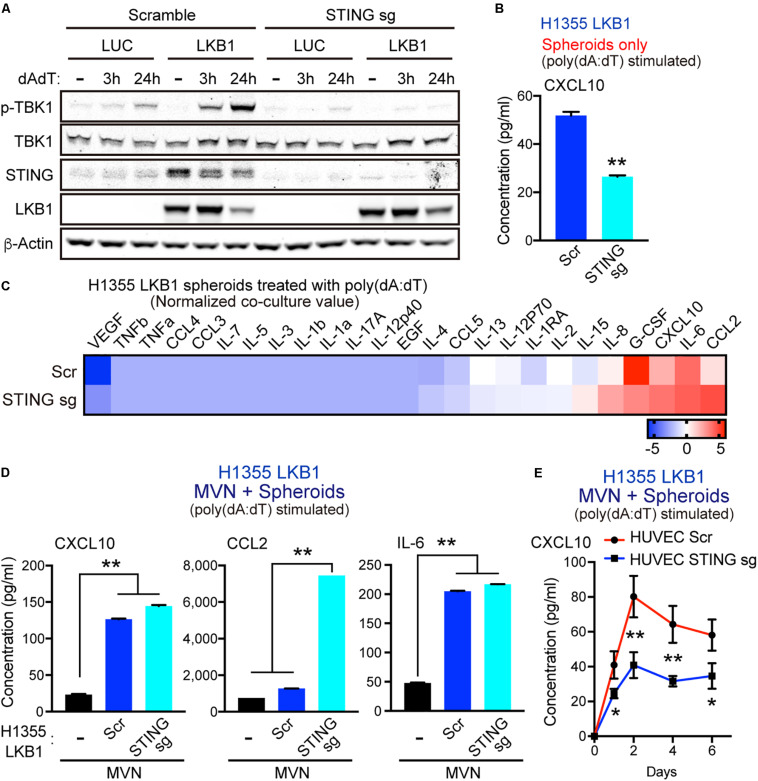
MVN co-culture enhances dsDNA-induced cytokines/chemokines even in the absence of tumor cell STING. **(A)** Immunoblot of the indicated proteins in H1355 cells transduced with LUC and LKB1 ± scramble (control sgRNA) or STING knockout (STING sgRNA) in 2D culture. **(B)** CXCL10 production in H1355-LKB1 spheroids after STING knockout, (*n* = 3 biological replicates). **(C)** Heat map of log2 fold change cytokine/chemokine profiles in conditioned medium (CM) after 7 days of 3D microfluidic culture of MVN with H1355 LUC and LKB1 spheroids with scramble or STING knockout. Spheroids were pre-treated with 1 μg/mL poly (dA:dT). **(D)** Absolute values of cytokine/chemokine release of CXCL10, CCL2, and IL-6 production. **(E)** ELISA of human CXCL10 over 6 days of 3D microfluidic culture of HUVEC STING knockout (STING sgRNA) or HUVEC scramble (control sgRNA) with H1355 LKB1 spheroids. Spheroids were pre-treated with 1 μg/mL poly (dA:dT). CM was collected after 1 day and every 2 days of 3D culture. *P* values were calculated by unpaired two tailed student *t*-test or one-way ANOVA followed by Tukey *post hoc* test; **P < 0.01, *0.01 < *P* < 0.05. Data shown as mean values, error bars ± SD.

To dissect how dsDNA activated tumor cells cooperate with MVNs to enhance cytokine release, we knocked out STING via CRISPR/CAS9 deletion in H1355 cells with or without LKB1 reconstitution. As expected, STING deletion prevented LKB1 restoration of STING expression and suppressed activation of TBK1, as measured by S172 phosphorylation after poly(dA:dT) treatment (Figure 3A). In consonance with this result, STING knockout in LKB1-reconstituted H1355 tumor spheroids also inhibited downstream production of CXCL10 (Figure 3B). We then conducted tumor spheroid/MVN co-culture experiments with STING knockout cell lines, hypothesizing that the cooperative increase in innate immune cytokine production seen in co-culture would be similarly blunted. Surprisingly, we observed comparable induction of cytokines/chemokines following poly(dA:dT) treatment in LKB1 reconstituted H1355 spheroids and MVNs even in the absence of tumor cell STING (Figure 3C). Specifically, we again observed CXCL10 and IL-6 production that was significantly higher in co-culture compared to the vasculature alone, regardless of tumor cell STING (Figure 3D). Production of CCL2 was further enhanced in LKB1 reconstituted spheroid co-culture without tumor cell STING activity as compared to STING intact spheroid co-culture or vasculature alone (Figure 3D). Interestingly, in contrast to STING knockout in LKB1-reconstituted H1355 spheroids, STING knockout in HUVECs consistently resulted in impaired production of CXCL10 in 3D MVN co-culture, despite only treating tumor cells with poly-(dA:dT) (Figure 3E). These data revealed that dsDNA-mediated cooperative induction of cytokines/chemokines in tumor spheroid/MVN co-culture does not rely on tumor cell STING, and instead might rely on endothelial STING activation.

### Endogenous Activation of TBK1 in the Tumor Vasculature

**FIGURE 4:**
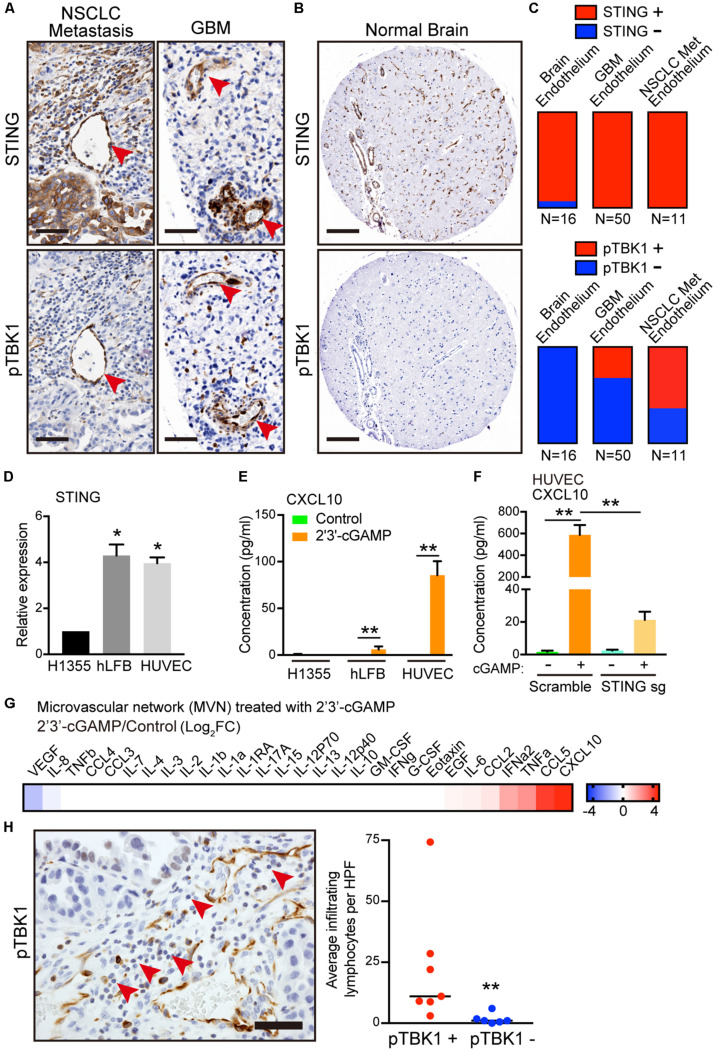
Activation of STING-TBK1 signaling in tumor vasculature and lymphocyte infiltration. **(A)** Representative IHC images from metastatic non-small cell lung cancer (NSCLC) and primary glioblastoma multiforme (GBM) patient brain tissue samples. Red arrows highlight STING (upper) and phospho-TBK1 (pTBK1, lower) staining. Scale bars, 50 μm. **(B)** Representative STING (upper) and pTBK1 (lower) IHC images from control patient brain tissue. Scale bars, 100 μm. **(C)** Endothelial STING and pTBK1 IHC was scored in a blinded manner for each sample on a binary scale based on the presence (positive, +) or absence (negative, -) of staining in cells surrounding the presumed endothelial lumen. **(D)** qRT-PCR of basal STING in parental H1355, hLFB, and HUVEC. **(F)** qRT-PCR of IFN-β and CXCL10 in HUVECs after exogenous 2′3′-cGAMP treatment. Cells were treated with 1 μg/mL 2′3′-cGAMP for 24 h. **(E)** ELISA of human CXCL10 levels in conditioned medium derived from parental H1355, hLFB, and HUVEC after exogenous 2′3′-cGAMP treatment (1 μg/mL) for 24 h. **(F)** ELISA of human CXCL10 levels in conditioned medium derived from HUVECs transduced with scramble (control sgRNA) or STING knockout (STING sgRNA), after exogenous 2′3′-cGAMP treatment (1 μg/mL) for 24 h. **(G)** Heat map of log2 fold change cytokine/chemokine profiles in conditioned medium (CM) after 7 days of 3D microfluidic culture of MVN treated with 1 μg/mL 2′3′-cGAMP treatment over MVN control. MVN were treated for 2 days after MVN formation. **(H)** Representative IHC image from metastatic non-small cell lung cancer (NSCLC) with pTBK1+ endothelial microvessels. Red arrows highlight infiltrating lymphocytes. Scale bars, 50 μm (left). Quantification of infiltrating lymphocytes per high power field (HPF) surrounding pTBK1+ or pTBK1- endothelial lumens, (right). *P* values were calculated by unpaired two tailed student *t*-test, one-way ANOVA, or two-way ANOVA followed by Tukey *post hoc* test; **P < 0.01, *0.01 < *P* < 0.05. Data shown as mean values, error bars ± SD, (*n* = 3 biological replicates).

To understand the potential relevance of this finding, we next examined expression of STING and activation of its immediate downstream target, phosphorylated TBK1 (pTBK1), across different tumor microenvironments in tissue microarrays (TMA) of patient samples. We focused on brain tumors and brain metastases, given the low baseline neuroinflammation and absent STING activation in normal brain. Notably, the most prominent and consistent areas of TBK1 activation in primary brain tumors and metastases were in cross-sections of tumor vasculature (Figures 4A,B). We scored STING expression and activation in a binary manner based on the presence or absence of STING and pTBK1 stain, in endothelial cells lining the lumen of blood vessels. Endothelial cells were identified histologically using the hematoxylin counterstain marking a circumferential layer of nuclei surrounding red blood cell fragments. IHC demonstrated endothelial STING staining in nearly every sample from either tumor- or normal brain-associated microvasculature (Figures 4A,C). Interestingly, pTBK1 IHC revealed specific activation of STING in the tumor endothelium of both primary GBM and metastatic NSCLC (Figures 4A,C), whereas normal brain samples stained uniformly negative for pTBK1 (Figures 4B,C).

Taken together with our *in vitro* co-culture findings, these data suggested a potential influence of tumor cells on activation of STING in the microvasculature, potentially in the form of tumor-derived 2′3′-cGAMP. Indeed, consistent with the human tumor data, both HUVECs and fibroblasts exhibited 4-fold higher expression of basal STING levels relative to H1355 (Figure 4D). Therefore, we next considered the effect of extracellular 2′3′-cGAMP on these different cell types and on established MVNs. Notably, HUVECs were significantly more sensitive to 2′3′-cGAMP treatment as compared to H1355 tumor cells and fibroblasts, producing significantly higher levels of IFN-β and especially CXCL10 [Figure 4E. As expected, STING knockout in HUVECs markedly reduced sensitivity to 2′3′-cGAMP treatment, especially as measured by CXCL10 production (Figure 4F and Supplementary Figures S3A,B)]. Cytokine/chemokine profiling analysis of established MVNs revealed that treatment of the vasculature with 1 μg/mL of 2′3′-cGAMP led to strong upregulation of multiple cytokines downstream of STING, such as CXCL10, CCL5, IFN-α, as well as CCL2 and IL-6 (Figure 4G and Supplementary Figure S2A).

To elucidate the potential impact of these findings on immune cell extravasation, we next quantified lymphocytes by morphologic criteria and examined their proximity to pTBK1+ versus pTBK1- vessels. We observed significantly greater perivascular lymphocyte infiltrates around pTBK1+ vessels, suggesting a potential relationship between activation of STING-TBK1 signaling in the tumor microvasculature and its ability to promote immune cell extravasation (Figure 4H). These findings thus prompted further examination of whether tumor derived 2′3′-cGAMP might directly activate STING in endothelial cells and prime the tumor vasculature for lymphocyte extravasation.

### Export of 2′3′-cGAMP by Tumor Cells Activates STING in Neighboring Endothelial Cells

**FIGURE 5:**
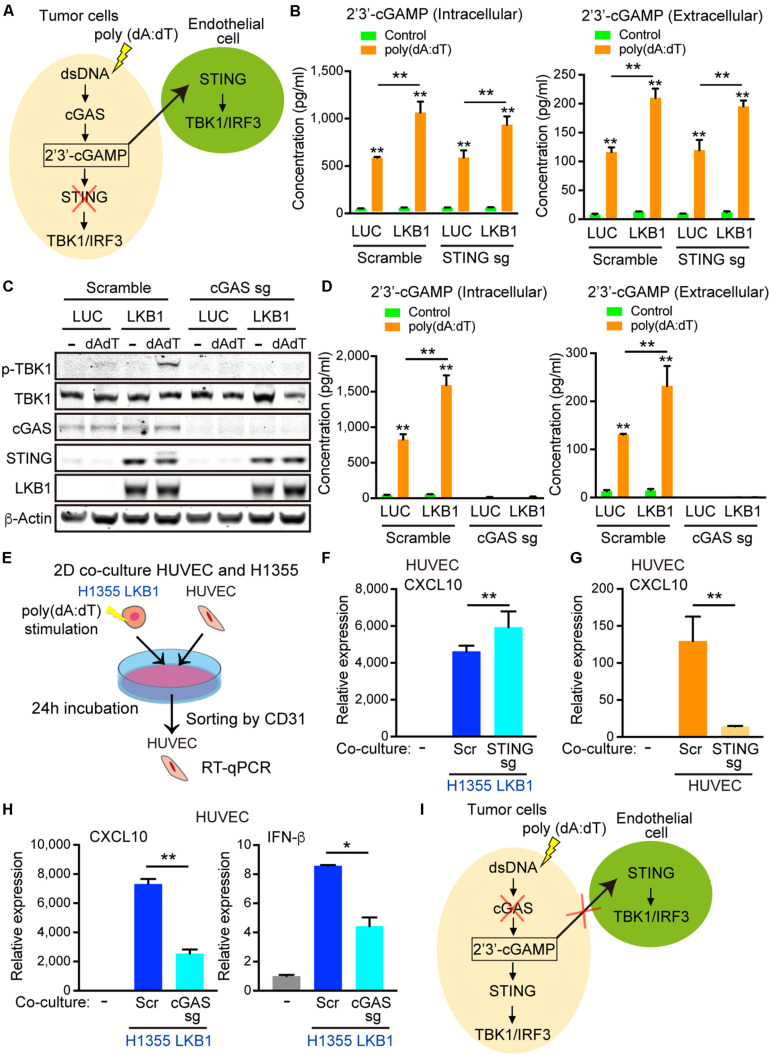
2′3′-cGAMP exported by cancer cells activates STING signaling in endothelial cells. **(A)** Schematic of cGAS-STING signaling in tumor cells after poly (dA:dT) stimulation and hypothesized export of 2′3′-cGAMP activating STING in the endothelial cells, which would be unaffected by tumor cell STING knockout. **(B)** Intracellular and extracelluar 2′3′-cGAMP ELISA of transduced LUC and LKB1 H1355 ± scramble or STING knockout. **(C)** Immunoblot of the indicated proteins in H1355 cells transduced with LUC and LKB1 with scramble or cGAS knockout (cGAS sgRNA) in 2-D culture. **(D)** Intracellular and extracellular 2′3′-cGAMP ELISA of transduced LUC and LKB1 H1355 with scramble or cGAS knockout. **(E)** Schematic of the 2-D co-culture experiment and sorting by CD31 + cells. **(F)** qRT-PCR of CXCL10 of HUVECs after 2-D co-culture with H1355 cells for 24 h with scramble or STING knockout. **(G)** qRT-PCR of CXCL10 of HUVECs scramble or STING knockout after 2-D co-culture with H1355 cells for 24 h. **(H)** qRT-PCR of CXCL10 and IFN-β of HUVECs after 2-D co-culture with H1355 cells for 24 h with scramble or cGAS knockout. **(I)** Schematic of cGAS-STING pathway in tumor cells after poly (dA:dT) stimulation demonstrating that silencing of cGAS impairs accumulation of 2′3′-cGAMP and export from the cells, limiting the STING activation in endothelial cells. *P* values were calculated by one-way ANOVA, or two-way ANOVA followed by Tukey *post hoc* test; **P < 0.01, *0.01 < *P* < 0.05. Data shown as mean values, error bars ± SD, (*n* = 3 biological replicates).

To determine whether 2′3′-cGAMP produced by cancer cells could activate STING/TBK1/IRF3 signaling in the neighboring tumor vasculature (Figure 5A), we first measured intracellular and extracellular 2′3′-cGAMP levels in H1355-LUC and H1355-LKB1 cells using the same concentration of double-stranded DNA used in co-culture experiments. Transfection of poly(dA:dT) not only increased levels of intracellular 2′3′-cGAMP, but also increased 2′3′-cGAMP levels in the media, which was significantly enhanced by LKB1 re-constitution (Figure 5B). As expected, knockout of STING failed to suppress either 2′3′-cGAMP intracellular production or extracellular export, regardless of LKB1 status (Figure 5B).

To directly assess whether 2′3′-cGAMP generated from tumor cells could activate STING in endothelial cells, we next generated cGAS knockout H1355 cells and treated them with poly(dA:dT). As expected, recognition of cytosolic DNA and subsequent downstream activation of STING was abolished in cGAS null H1355-LUC and H1355-LKB1 cell lines, even after the de-repression of STING following LKB1 re-constitution (Figure 5C). Consistent with this, intracellular 2′3′-cGAMP production was significantly diminished, and there were virtually undetectable levels of 2′3′-cGAMP in the media from the cGAS knockout line (Figure 5D). We next investigated the impact of co-culturing poly(dA:dT) transfected H1355-LKB1 cells following STING or cGAS knockout with HUVECs, sorting CD31 positive cells to measure HUVEC specific cytokines/chemokines (Figure 5E). Consistent with our results in 3D culture, dsDNA stimulation of H1355-LKB1 reconstituted cells potently activated CXCL10 as well as IFN-β expression in HUVECs, even following STING knockout in H1355-LKB1 cells (Figure 5F). Meanwhile, CXCL10 production was dramatically reduced by STING knockout in HUVECs co-cultured with H1355-LKB1 cells, further substantiating a direct contribution of endothelial cell STING to induction of CXCL10 secretion in the 3D microfluidic culture system (Figure 5G). Importantly, we also observed that cancer cell cGAS deletion significantly decreased expression of both CXCL10 and IFN-β in co-cultured HUVECs, providing direct evidence that ablation of 2′3′-cGAMP export from tumor cells suppresses STING signaling in endothelial cells (Figures 5H,I). Of note, expression of CXCL10 and IFN-β in HUVECs was not completely abolished following cGAS knockout, suggesting the potential for additional paracrine mediators of this response downstream of alternate dsDNA sensors. Regardless, these data confirm that cGAS-driven 2′3′-cGAMP export from cancer cells is directly involved in activation of STING signaling in neighboring endothelial cells, prompting us to examine whether it might play additional roles in priming the vasculature to promote immune cell recruitment.

### 2′3′-cGAMP and IFN-β Prime the Endothelium for Immune Cell Extravasation

**FIGURE 6:**
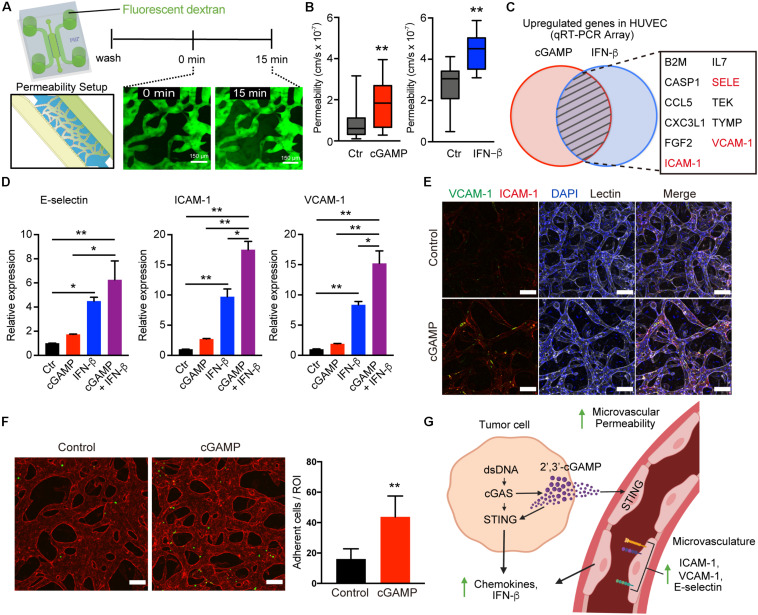
2′3′-cGAMP and IFN-β promote vascular permeability and upregulation of adhesion molecules. **(A)** Schematic of permeability experiments and analysis. Dextran dye was injected, and image stacks were captured at time 0 and at 15 min. **(B)** Permeability coefficients for different conditions (control, cGAMP, IFN-β), (*n* = 9 biological replicates). **(C)** Venn diagram of top genes upregulated in HUVEC after treatment with 2′3′-cGAMP or IFN-β. **(D)** qRT-PCR of E-selectin, ICAM-1, VCAM-1 in HUVEC treated with 2′3′-cGAMP, IFN-β, or combination of 2′3′-cGAMP + IFN-β (*n* = 3 biological replicates). **(E)** Confocal images of microvasculature treated ± 2′3′-cGAMP immunostained for ICAM-1, VCAM-1, and lectin. Scale bars, 100 μm. **(F)** Confocal images of microvasculature (red) treated ± 2′-3′-cGAMP and Jurkat cells (green) (left). Quantification of the number of Jurkat cells adherent to microvasculature per Region of Interest (ROI) (right). Scale bars, 100 μm. **(G)** Schematic of tumor-derived cGAMP (via cGAS) and IFN-β (via STING) influencing the vascular permeability, adhesion molecules, and chemokines. Schematic was created with BioRender. *P* values were calculated by unpaired two tailed student *t*-test or one-way ANOVA followed by Tukey *post hoc* test; **P < 0.01, *0.01 < *P* < 0.05. Data shown as mean values, error bars ± SD.

To determine whether 2′3′-cGAMP might alter the vasculature to promote immune cell recruitment, we first assessed its impact on microvascular permeability. Permeability of MVNs in microfluidic culture can be measured via flux of a fluorescent tracer from the luminal compartment of the vasculature to the interstitial space, as recently described (21). We assessed the effects of 2′3′-cGAMP or downstream type I IFN, which can also impact tumor vasculature (29), and observed that both 2′3′-cGAMP and IFN-β treatment individually enhanced permeability of the 3-d MVNs (Figures 6A,B). We next tested a quantitative PCR array of endothelial activation markers to identify whether expression of specific adhesion molecules or other related genes are also upregulated after treatment of endothelial cells with 2′3′-cGAMP or IFN-β. Notably, analysis of this list to identify common targets shared by both 2′3′cGAMP and IFN-β treatment revealed prominent upregulation of multiple genes involved in T cell trafficking, including E-selectin, ICAM-1, and VCAM-1 (Figure 6C). We therefore examined expression of these specific genes in HUVECs in response to 2′3′-cGAMP, IFN-β, or the combination. Indeed, all three genes were significantly induced by 2′3′-cGAMP or IFN-β, treatment, and further increased by the combination (Figure 6D). In contrast, we observed negligible changes in expression of genes involved in tight junctions, including ZO-1, Occludin and Claudin-5 (Supplementary Figure S4C), suggesting that the observed changes in vascular permeability are likely occur at the post-transcriptional level. We therefore focused on differences in expression of adhesion molecules in established MVNs in 3D microfluidic devices. Treatment with 2′3′-cGAMP and/or IFN-β increased expression of membrane-bound ICAM-1 and VCAM-1 on MVNs by immunofluorescence (Figure 6E and Supplementary Figure S4B). To determine the functional consequences of this adhesion molecule upregulation, we perfused Jurkat T cells through the MVNs and observed significantly increased attachement of Jurkat cells to the endothelial walls of the microvasculature in the presence of 2′3′cGAMP pre-treatment (Figure 6F). Taken together, these findings reveal that tumor derived 2′3′-cGAMP not only amplifies cytokine production in the adjacent vasculature, but also increases permeability and upregulates adhesion molecules that can facilitate T cell escape (30, 31) (Figure 6G).

## Discussion

Here, we utilize a novel microfluidic co-culture system to probe the impact of LKB1 inactivation on cancer cell intrinsic cGAS-STING signaling in relationship to the microvasculature, a critical gatekeeper of immune cell extravasation. Cytokine profiling of conditioned media from the co-culture of 3-D MVNs with tumor spheroids revealed a cooperative production of multiple chemo-attractants downstream of STING such as CXCL10, CCL5, and CCL2, which was surprisingly independent of cancer cell STING. Furthermore, examination of primary tumors and metastases from patients demonstrated STING-TBK1 activation principally in the tumor vasculature, in contrast to the healthy vascular endothelium. These data suggest paracrine signaling from cancer cells to promote STING activation in adjacent vasculature. Further investigation revealed that 2′3′-cGAMP preferentially activates endothelial cells, that LKB1 reconstitution enhances 2′3′-cGAMP export, and that cancer cell-intrinsic cGAS activity contributes directly to endothelial cell activation. Finally, we show that STING activation in the endothelium by 2′3′-cGAMP can be further enhanced by downstream type 1 IFN, resulting in functional changes to the vasculature that favor immune cell extravasation.

Several recent studies have highlighted an emerging role of cancer cell derived 2′3′-cGAMP in the tumor microenvironment (TME). For example, tumor cells have been shown to transfer 2′3′-cGAMP to astrocytes directly via gap junctions, activating NF-κB signaling and promoting adaptation of the TME to facilitate brain metastasis (7). More recently, multiple groups have unveiled a direct role for exported cancer cell 2′3′-cGAMP in activating anti-tumor immunity. For example, using murine syngeneic cancer lines, tumor-derived 2′3′-cGAMP was shown to activate STING in neighboring host cells to produce type I interferons in the TME and subsequently prime NK cells for tumor cell lysis (8). In two very recent studies, the role of extracellular 2′3′-cGAMP as an immunotransmitter has been further solidified. Inhibition of tumor-associated macrophages has been shown to drive type 1 interferon production in the TME via accumulation of extracellular cancer cell derived 2′3′-cGAMP (13). Furthermore, inhibition of the 2′3′-cGAMP hydrolase ENPP1 was reported to enhance export and accumulation in the media both at steady state and following DNA damage, promoting tumor associated immune infiltration (14). However, to date, a role for endogenous 2′3′-cGAMP in promoting vascular activation, a pre-requisite for this immune cell influx into tumors, has yet to be described.

Intratumoral administration of 2′3′-cGAMP or other cyclic dinucleotide STING agonists promotes therapeutic immune response in several mouse tumor models dependent on host STING but only partially dependent on host T-cell activity (32). Furthermore, tumor endothelial cells have been shown to represent the major source of type I interferon production in the TME following intratumoral injection of 3′3′-cGAMP in the B16F10 melanoma model (15). We now provide evidence that tumor-derived 2′3′-cGAMP can activate endothelial STING, and that 2′3′-cGAMP and downstream IFN-β enhance vascular permeability. Immune cell effectors must receive cues to adhere and extravasate (33) from the vasculature in order to enter the tumor microenvironment and we observed that 2′3′-cGAMP and IFN-β co-operate to upregulate E-selectin, ICAM-1, and VCAM-1. In this regard, endogenous tumor-derived 2′3′-cGAMP could play a gatekeeper role in determining the ability of immune cells to infiltrate by regulating vascular expression of ICAM-1 (27) and VCAM-1 (34), though this possibility needs to be further validated *in vivo*, including animal studies. Regardless, our data suggests that, in addition to silencing STING, downregulation of 2′3′-cGAMP through mutations such as LKB1 could also promote immune cell exclusion from tumors.

STK11/LKB1 mutation has been identified as a main driver of anti-PD1 resistance in KRAS-mutant lung adenocarcinoma, and loss of LKB1 has been linked to decreased numbers and function of T cells in the TME (5, 24, 35). We have previously reported *in vivo* quantitative IHC data from patient biopsies with KL tumors showing that T cells were retained in the stroma rather than infiltrating the cancer epithelium, suggesting that LKB1 is important for T cell recruitment (5). Our new data offer a more refined explanation for the phenomenon of defective T cell chemotaxis seen in the KL TME. The recognition of tumor-derived 2′3′-cGAMP and resultant aberrant STING activation by neighboring tumor vasculature, along with attenuated vascular activation and decreased production of T cell chemokines such as CXCL10 by the KL cancer cells, may contribute to the disruption of T cell chemotaxis and resistance to anti-PD1 treatment in the KL TME. We also note that our IHC studies relied on basal tumors unexposed to DNA damaging agents, as evidenced by lack of robust tumor cell pTBK1 staining. Thus, active therapy with agents that enhance endogenous tumor cGAMP production either through non-specific DNA damage (chemotherapy or radiation) or more targeted approaches such as TREX1 or ENPP1 inhibition, are likely to prime even more robust vascular activation, T cell recruitment and infiltration in the TME. Additionally, while we focused on tumor-derived 2′3′-cGAMP as the source of vascular activation in our co-culture system, extravasated antigen presenting cells in the TME could represent another source of extracellular 2′3′-cGAMP. In addition, macrophages are known to produce IFN-β in response to extracellular 2′3′-cGAMP, which could also contribute to vascular activation via paracrine signaling (36). Finally, although we did not observe robust cytokine production from fibroblasts in response to extracellular 2′3′-cGAMP in our systems, fibroblasts are critical in the formation of a vascularized tumor niche and it remains to be seen how extracellular 2′3′-cGAMP may influence the secretome from cancer-associated fibroblasts (37).

More generally, this study demonstrates that developing more representative physiological cell culture models of the TME can elucidate important aspects of innate immune signaling that cannot be studied using traditional *in vitro* systems (38). Indeed, microfluidic technologies help develop new tools for cancer diagnosis and treatment. Important future directions to enhance the physiologic relevance of the microfluidic co-culture system include introduction of continuous perfusion to mimic blood microcirculation and enable modeling of immune cell trafficking in a long-term culture system. Furthermore, advances in *ex vivo* modeling may allow long-term culture of patient-derived tumor samples to enable a personalized medicine approach to study drug or immune cell penetration in patient-specific tumor niches (18, 20).

In summary, our data expands upon the increasingly recognized role of endogenous tumor-derived 2′3′-cGAMP in the TME (39), revealing a novel function in vascular activation and immune escape following LKB1 loss. They also suggest that DNA damaging agents or targeted therapeutics that increase endogenous 2′3′-cGAMP production may act to enhance vascular activation and improve immune infiltration and subsequent response to PD-1 blockade in these treatment refractory cancers.

## Author’s Note

While critical for tumor immune evasion, the phenomenon of immune cell exclusion remains incompletely understood. Using KRAS-LKB1 mutant lung cancer as a model, we demonstrate that tumor cell cGAS-STING not only regulates chemokine-mediated immune cell recruitment, but also directly influences the gatekeeper function of tumor vasculature via extracellular transfer of 2′3′-cGAMP.

## Data Availability Statement

The data are available upon requests to the corresponding authors (DB, SK, and RK).

## Author Contributions

MC, SKS, SES, RK, SK, and DB designed the research and wrote the manuscript. MC, SKS, SES, SK, EK, EI, IC, RY, and TeT performed and supervised biological and cellular studies. MC, SES, TO, and BP designed and prepared the microfluidic device. MC, SKS, and SES performed cell culture in microfluidic devices. SES, MC, TO, and SL performed permeability measurements, confocal imaging, and computational analyses. EK, NM, EI, and CP obtained samples and performed or supervised immunohistochemistry. SKS, TrT, SH, SG, SK, and RY performed multiplexed arrays, immunoblots, ELISA, and RT-PCR. MC, SKS, SES, SK, EK, NM, and SL analyzed the data. CP, VC, RK, SK, and DB supervised the project. All authors read and approved the manuscript.

## Conflict of Interest

RK is co-founder and has a significant financial interest in AIM Biotech, a company that manufactures microfluidic systems, and has received research funding from Amgen and Biogen. DB and CP are co-founders and scientific advisory members of Xsphera Biosciences. DB is a consultant for N-of-One/Qiagen, a scientific advisory board member of Tango Therapeutics, and has received research funding from Bristol-Myers Squibb, Novartis, Lilly Oncology, and Gilead Sciences. The remaining authors declare that the research was conducted in the absence of any commercial or financial relationships that could be construed as a potential conflict of interest.

## Footnotes

1 https://portals.broadinstitute.org/gpp/public/analysis-tools/sgrna-design

## Supplementary Material

FIGURE S1 LKB1 reconstitution in 3D microfluidic spheroid culture. **(A)** Immunoblot of the indicated proteins in H1355 cells transduced with LUC and LKB1. **(B)** log10 of the absolute values of cytokine release corresponding to the heatmap in Figure 1D from 3D microfluidic culture H1355 LUC spheroids and H1355 LKB1-reconstituted spheroids. **(C)** Schematic of human lung fibroblasts (hLFB) and human umbilical vein endothelial cells (HUVEC) in a 3D microfluidic device within a collagen/fibrin hydrogel for 7 days. Confocal images of microvasculature (MVN) immunostained for F-actin (red) and CD31 (green). Scale bar, 150 μm. **(D)** log10 of the absolute values of cytokine production corresponding to the heatmap in Figure 1D from 3D microfluidic culture of microvascular networks (MVN) alone.

FIGURE S2 KL-cGAMP export and sensitivity of HUVECs to cGAMP. **(A)** Log10 of the absolute values of cytokine production corresponding to the heatmap in Figure 4G, from 3D microfluidic culture of MVN treated with 1 μg/mL 2′3′-cGAMP treatment over MVN control.

FIGURE S3 Insensitivity of STING knockout HUVEC to 2′3′-cGAMP. **(A)** Immunoblot of the indicated proteins in HUVEC transduced with scramble (control sgRNA) or STING knockout (STING sgRNA). **(B)** qRT-PCR of CXCL10 and IFN-β of HUVECs transduced with scramble (control sgRNA) or STING knockout (STING sgRNA), after exogenous 2′3′-cGAMP treatment (1 μg/mL) for 24 h. *P* values were calculated by two-way ANOVA followed by Tukey *post hoc* test; ^∗∗^*P* < 0.01. Data shown as mean values, error bars ±SD.

FIGURE S4 cGAMP/IFN-β affects adhesion molecules. **(A)** Upregulated genes from HUVEC treated with 2′3′-cGAMP or IFN-β. **(B)** Immunostaining of ICAM-1 and VCAM-1 in networks treated with IFN-β (100 ng/ml) or in combination with 2′3′-cGAMP. Scale bars, 100 μm. **(C)** qRT-PCR of ZO-1, Occludin and Claudin-5 in HUVEC treated with 2′3′-cGAMP, IFN-β, or combination of 2′3′-cGAMP + IFN-β.

FIGURE S5 Design of microfluidic devices. **(A)** The 3D cell culture chip (AIM Biotech) is shown with three independent microfluidic chambers (referred to as “device”) per chip, Each device contains a center gel region with posts separating the gel region from the anti-parallel side channels. **(B,C)** Custom PDMS microfluidic devices were designed using Autocad (Autodesk) and are comprised of a central gel channel, two medium channels and four reservoirs. Devices were bonded to glass coverslips.
