# Supplementary figures and images for: Tumor-Derived cGAMP Regulates Activation of the Vasculature
Source: Front Immunol. 2020 Sep 4;11:2090. doi: 10.3389/fimmu.2020.02090 (PMC7507350; doi:10.3389/fimmu.2020.02090)

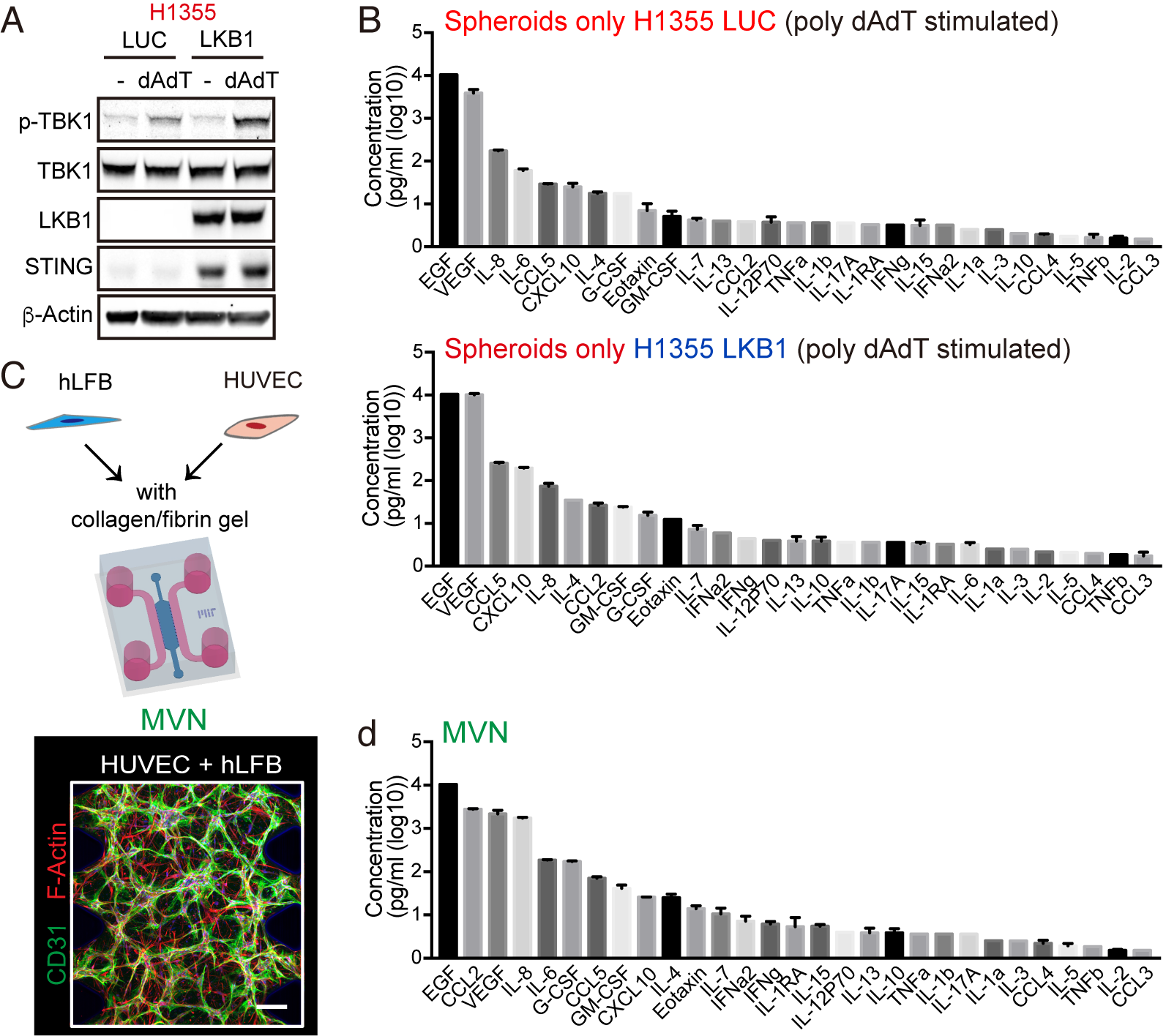

Supplement: FIGURE S1 — LKB1 reconstitution in 3D microfluidic spheroid culture. (A) Immunoblot of the indicated proteins in H1355 cells transduced with LUC and LKB1. (B) log10 of the absolute values of cytokine release corresponding to the heatmap in Figure 1D from 3D microfluidic culture H1355 LUC spheroids and H1355 LKB1-reconstituted spheroids. (C) Schematic of human lung fibroblasts (hLFB) and human umbilical vein endothelial cells (HUVEC) in a 3D microfluidic device within a collagen/fibrin hydrogel for 7 days. Confocal images of microvasculature (MVN) immunostained for F-actin (red) and CD31 (green). Scale bar, 150 μm. (D) log10 of the absolute values of cytokine production corresponding to the heatmap in Figure 1D from 3D microfluidic culture of microvascular networks (MVN) alone. [file Image_1.TIF]

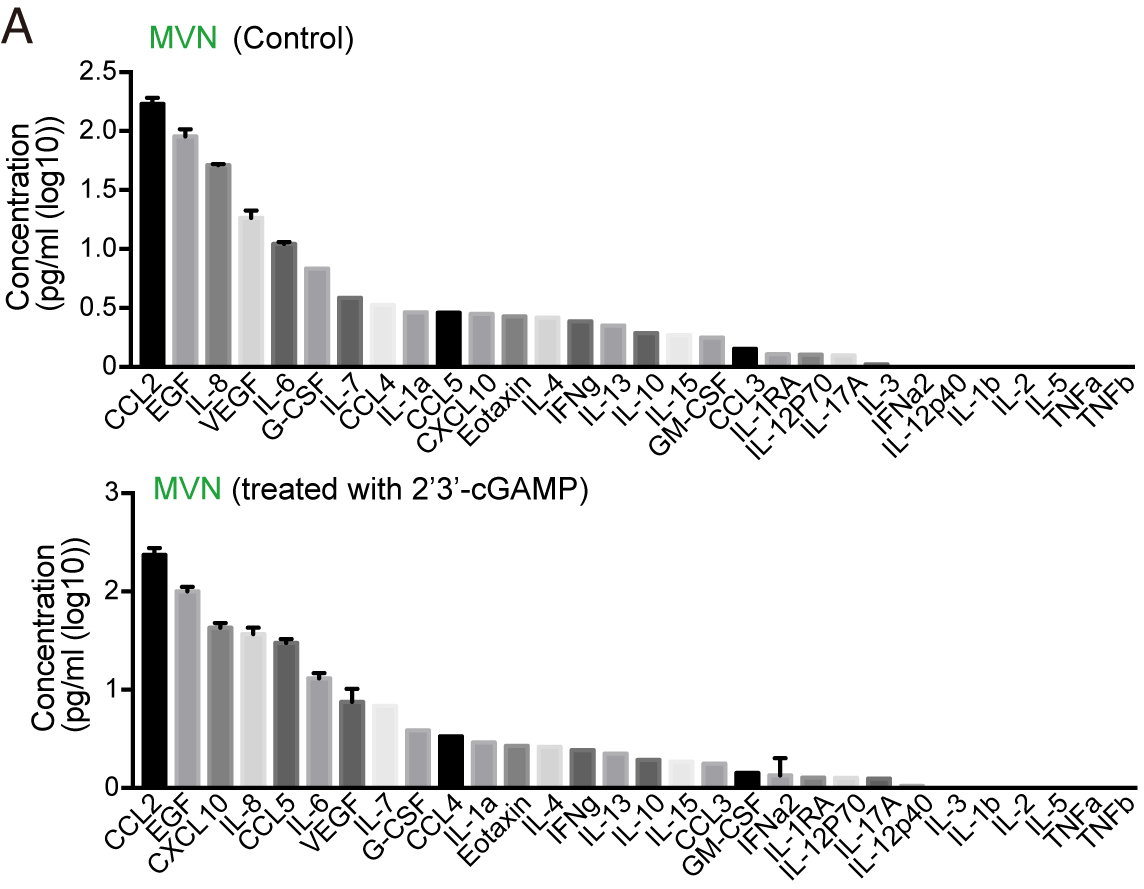

Supplement: FIGURE S2 — KL-cGAMP export and sensitivity of HUVECs to cGAMP. (A) Log10 of the absolute values of cytokine production corresponding to the heatmap in Figure 4G, from 3D microfluidic culture of MVN treated with 1 μg/mL 2′3′-cGAMP treatment over MVN control. [file Image_2.TIF]

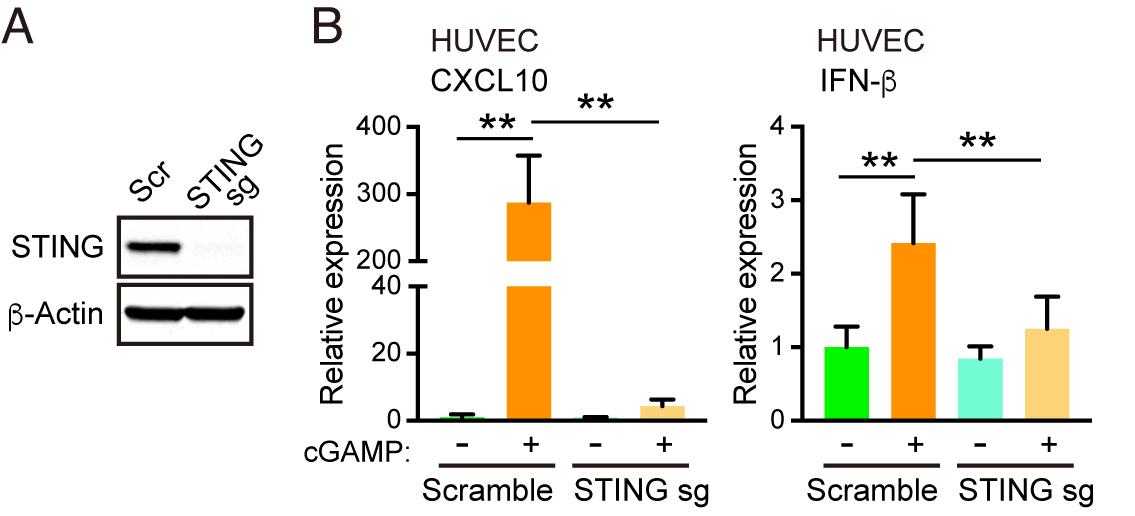

Supplement: FIGURE S3 — Insensitivity of STING knockout HUVEC to 2′3′-cGAMP. (A) Immunoblot of the indicated proteins in HUVEC transduced with scramble (control sgRNA) or STING knockout (STING sgRNA). (B) qRT-PCR of CXCL10 and IFN-β of HUVECs transduced with scramble (control sgRNA) or STING knockout (STING sgRNA), after exogenous 2′3′-cGAMP treatment (1 μg/mL) for 24 h. P values were calculated by two-way ANOVA followed by Tukey post hoc test; ∗∗P < 0.01. Data shown as mean values, error bars ±SD. [file Image_3.TIF]

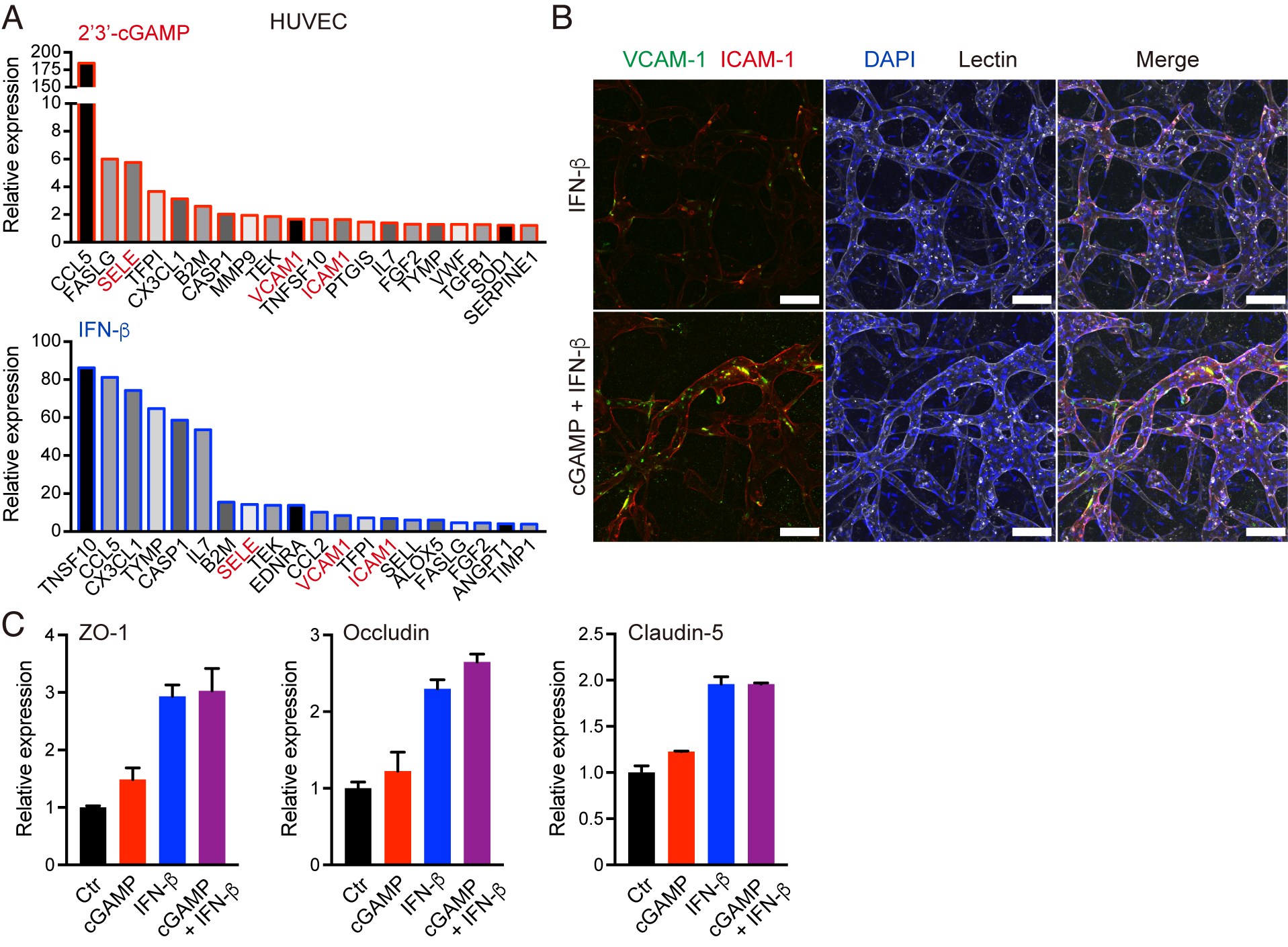

Supplement: FIGURE S4 — cGAMP/IFN-β affects adhesion molecules. (A) Upregulated genes from HUVEC treated with 2′3′-cGAMP or IFN-β. (B) Immunostaining of ICAM-1 and VCAM-1 in networks treated with IFN-β (100 ng/ml) or in combination with 2′3′-cGAMP. Scale bars, 100 μm. (C) qRT-PCR of ZO-1, Occludin and Claudin-5 in HUVEC treated with 2′3′-cGAMP, IFN-β, or combination of 2′3′-cGAMP + IFN-β. [file Image_4.TIF]

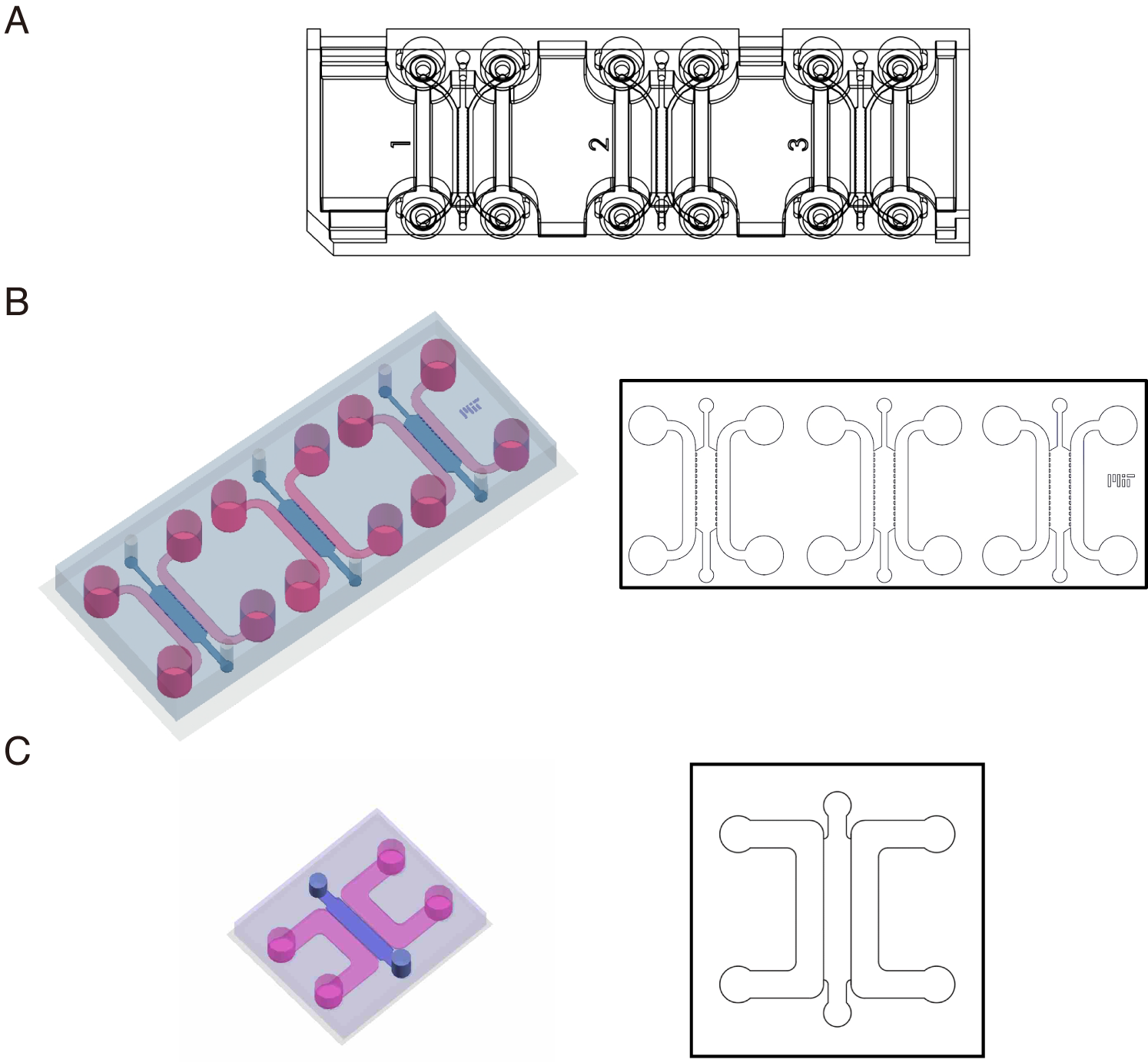

Supplement: FIGURE S5 — Design of microfluidic devices. (A) The 3D cell culture chip (AIM Biotech) is shown with three independent microfluidic chambers (referred to as “device”) per chip, Each device contains a center gel region with posts separating the gel region from the anti-parallel side channels. (B,C) Custom PDMS microfluidic devices were designed using Autocad (Autodesk) and are comprised of a central gel channel, two medium channels and four reservoirs. Devices were bonded to glass coverslips. [file Image_5.TIF]
